# Role of the *Yersinia pestis* Yersiniabactin Iron Acquisition System in the Incidence of Flea-Borne Plague

**DOI:** 10.1371/journal.pone.0014379

**Published:** 2010-12-17

**Authors:** Florent Sebbane, Clayton Jarrett, Donald Gardner, Daniel Long, B. Joseph Hinnebusch

**Affiliations:** 1 Equipe Peste et Yersinia pestis, Centre d'Infection et d'Immunité de Lille, Institut Pasteur de Lille, Lille, France; 2 INSERM U1019, Lille, France; 3 CNRS UMR8204, Lille, France; 4 Université Lille Nord de France, Lille, France; 5 UDSL, Centre d'Infection et d'Immunité de Lille, Lille, France; 6 Laboratory of Zoonotic Pathogens and Veterinary Pathology Section, Rocky Mountain Laboratories, National Institute of Allergy and Infectious Diseases, National Institutes of Health, Hamilton, Montana, United States of America; 7 Veterinary Pathology Section, Rocky Mountain Laboratories, National Institute of Allergy and Infectious Diseases, National Institutes of Health, Hamilton, Montana, United States of America; East Carolina University School of Medicine, United States of America

## Abstract

Plague is a flea-borne zoonosis caused by the bacterium *Yersinia pestis*. *Y. pestis* mutants lacking the yersiniabactin (Ybt) siderophore-based iron transport system are avirulent when inoculated intradermally but fully virulent when inoculated intravenously in mice. Presumably, Ybt is required to provide sufficient iron at the peripheral injection site, suggesting that Ybt would be an essential virulence factor for flea-borne plague. Here, using a flea-to-mouse transmission model, we show that a *Y. pestis* strain lacking the Ybt system causes fatal plague at low incidence when transmitted by fleas. Bacteriology and histology analyses revealed that a Ybt-negative strain caused only primary septicemic plague and atypical bubonic plague instead of the typical bubonic form of disease. The results provide new evidence that primary septicemic plague is a distinct clinical entity and suggest that unusual forms of plague may be caused by atypical *Y. pestis* strains.

## Introduction

*Yersinia pestis* is usually transmitted by infected fleas and produces bubonic plague, characterized by a painful, swollen lymph node, the bubo [1]. Bubonic plague progresses rapidly to a life-threatening septicemia, but septicemia without a prior bubonic phase (primary septicemic plague), may also result from direct injection of plague bacilli into a blood vessel during the flea bloodmeal [2]. Other less common clinical presentations that can follow flea-borne transmission include pestis minor (a benign form of bubonic plague) and carbuncular plague with or without palpable buboes [1], [3]. These rare forms of plague have not been attributed to atypical strains of *Y. pestis.* However, atypical strains have been isolated from around the world and it remains unclear whether these isolates produce one or another form of plague.

Typical *Y. pestis* strains form red colonies (pigmented or Pgm^+^) after growth at ≤34°C on media containing Congo red, but white colonies (Pgm^–^) may be isolated at a frequency of 10^−4^ [4]. Most spontaneous Pgm^–^ mutants result from the deletion of a 102-kb chromosomal region termed the *pgm* locus [5], [6]. This locus includes the haemin storage operon (*hmsHFRS*) which is essential for the pigmentation phenotype and for the production of a biofilm in the flea gut that can block normal blood feeding; the blockage of the flea's digestive tract is considered to be an important process for flea-borne transmission [7]. The *pgm* locus also contains the Yersinia high-pathogenicity island (HPI), which carries among other genes the *irp1-irp2-ybtU-ybtT-ybtE,* the *ybtP-ybtQ-ybtX-ybtS* and the *psn* loci that encode the yersiniabactin (Ybt) siderophore-based iron acquisition and transport system. The *irp* genes encode the high molecular weight proteins (HMWP) 1 and 2 which act in concert with YbtU, YbtE, YbtS and probably YbtT to synthesize the Ybt siderophore [8]. Ybt is secreted, acquires iron from transferrin and lactoferrin in host tissues, then is transported back into *Y. pestis* by the TonB-dependent outer membrane receptor Psn and the inner membrane ABC-transporter YbtP-YbtQ. A critical role of the Ybt system in bubonic plague is indicated by the fact that Ybt^–^ *Y. pestis* strains are essentially avirulent by the subcutaneous inoculation route that mimics the flea bite, although these strains retain complete or nearly complete virulence when inoculated intravenously [5], [9], [10], [11]. Presumably, Ybt is required to provide sufficient iron at the peripheral injection site, in the draining lymphatic system, and/or in the lymph nodes, suggesting that Ybt would be an essential virulence factor for flea-borne bubonic plague.

Despite the importance of the Hms and Ybt system for flea-borne transmission and for disease in bubonic plague models, respectively, the *pgm* locus is subject to complete or partial loss at relatively high frequency by genomic rearrangements; and Pgm^–^ Ybt^–^ and Pgm^–^Ybt^+^ strains from natural plague foci have been described [5], [12]. Furthermore, human cases of plague have been associated with non-pigmented strains [12]. Altogether, the data prompted us to assess the role of the Ybt system in plague epidemiology and pathogenesis in the natural context of transmission by flea bite.

## Methods

The fully virulent *Y. pestis* 195/P strain which was originally isolated from a patient with pneumonic plague [13] and an isogenic *irp2* mutant with an in-frame deletion of bases 242 to 5721 were used in this study. The *irp2* mutant was generated by allelic exchange using the suicide plasmid pCVD442 and verified by sequencing. Bacteria cultured overnight in Luria broth at 21°C without aeration were quantified by using a Petroff-Hausser bacterial counting chamber, diluted in PBS and inoculated intravenously into the tail vein or intradermally in the upper right thigh to groups of 8–10 week-old female RML Swiss-Webster mice.

A flea-borne transmission model was used to determine *Y. pestis* infectivity after challenge by flea bite [2]. *Xenopsylla cheopis* rat fleas were allowed to feed on heparanized mouse blood containing wild-type *Y. pestis* or an isogenic *irp2* mutant, using an artificial feeding device, and maintained as previously described [7]. Beginning 13 days after their infectious bloodmeal (the time required for *Y. pestis* to block *X. cheopis*), 49 to 115 fleas were applied to a restrained mouse and allowed to feed for 60 min. Immediately after the challenge, the fleas were examined individually under a dissecting microscope to determine how many infective (blocked) fleas had bitten each mouse [7]. Mice that did not develop any symptoms within 5 days following a challenge were re-challenged until the cumulative number of bites from blocked fleas was high enough to consider that a successful transmission occurred [2], [14]. Mice received one to six different sequential challenges. Challenged animals were observed at least three times daily for three weeks and euthanized upon signs of terminal plague (evidence of lethargy, hunched posture, and reluctance to respond to external stimuli) [15]. Bacterial load in the spleen and blood was determined by colony-forming unit (CFU) count. Hematoxylin and eosin (H&E) and immunohistochemical (IHC) staining to detect *Y. pestis* [15] was performed on formalin-fixed inguinal lymph node sections.

### Ethics Statement

All animal experiments were approved by the Rocky Mountain Laboratories, National Institute of Allergy and Infectious Diseases, National Institutes of Health Biosafety and Animal Care and Use Committees in accordance with National Institutes of Health guidelines (animal protocol number 05-37).

## Results

**Figure 1:**
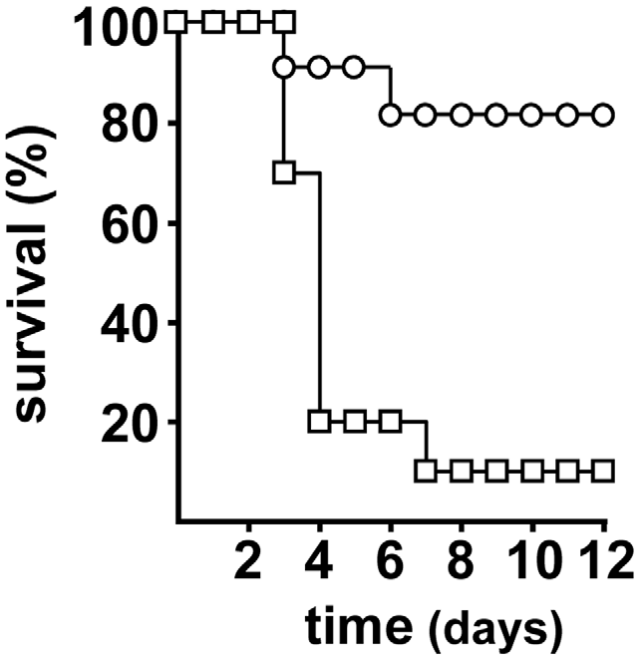
Effect of the *Y. pestis* Ybt on transmission by fleas. Incidence and time to terminal disease in mice bitten by fleas infected with *Y. pestis* wild type (open squares) or the Δ*irp2* mutant (open circles).

The Ybt system is not required for *Y. pestis* to colonize and block fleas [7]. Therefore, it was possible to compare the virulence of the wild-type *Y. pestis* strain and an isogenic Ybt-negative mutant after natural transmission by infected *X. cheopis* rat fleas. We first tested the virulence of our Ybt^–^ mutant after needle-inoculation of cultured bacteria. The LD50 of the mutant was 10 CFU after intravenous (IV) injection, but >10^5^ CFU after intradermal (ID) inoculation, similar to what has been reported previously for other Ybt^–^ *Y. pestis* strains [5], [9], [10], [11]. Next, we challenged mice using our flea-borne transmission model. Nine of the ten mice bitten by fleas infected with the wild-type strain developed terminal plague, eight of them within the first four days after challenge (Figure 1 and Table 1). Interestingly, although the Ybt^–^ mutant was highly attenuated when inoculated intradermally by needle, fleabites from fleas infected with this mutant produced terminal disease in two of ten mice, at 3 and 6 days post-challenge (Figure 1 and Table 1). Thus, the Ybt iron acquisition system is not essential to produce plague after fleabite transmission, although the incidence of disease was significantly lower in mice challenged by fleas infected with the *irp2* mutant (*P* = 0.001 by log-rank test).

**Table 1:** Disease outcome in mice bitten by fleas infected with wild-type or *irp2 Y. pestis*.

	Fleas infected with:						
	*Y. pestis* wild-type			*Y. pestis* Δ*irp2*			
Mouse	Infective flea bites* (*number of challenges*)	Time to terminal disease (days)	Outcome†	Mouse	Infective flea bites* (*number of challenges*)	Time to terminal disease (days)	Outcome†
1	1 (*1*)	4	B	11	7 (*3*)	6	S; a/B
2	1 (*1*)	7	B	12	5 (*3*)	3	S
3	2 (*1*)	4	B	13	7 (*4*)	-	-
4	4 (*1*)	4	B	14	9 (*6*)	-	-
5	5 (*1*)	3	B	15	6 (*5*)	-	-
6	6 (*1*)	3	B	16	6 (*5*)	-	-
7	3 (*1*)	4	S	17	6 (*5*)	-	-
8	8 (*2*)	3	S	18	8 (*5*)	-	-
9	9 (*2*)	4	S	19	5 (*6*)	-	-
10	11 (*2*)	-	-	20	9 (*4*)	-	-
Median	4.5 (*1*)	4			6.5 (*5*)	4.5	

*Cumulative number of bites from blocked fleas.

† B and a/B, typical and atypical bubonic plague respectively; S, primary septicemic plague; -, no disease.

**Figure 2:**
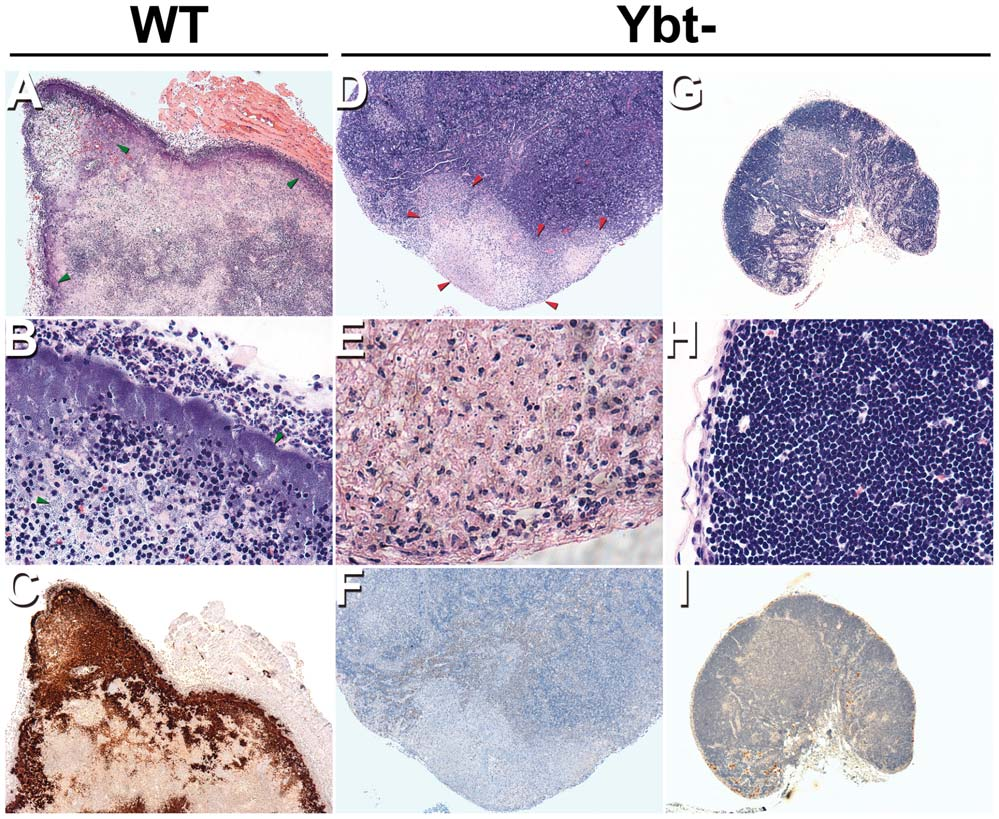
Lymph node histology of mice with terminal plague following flea-borne transmission of wild-type and Δ*irp2 Y. pestis.* Lymph node sections from mice infected with the wild-type strain (A to C) or with the Δ*irp2* strain (D to I) were strained by H&E (A, B, D, E, G and H) or by IHC using *Y. pestis*-specific antibody (C, F and I). Panels D, E and F and the panels G, H and I are photos of the lymph node from mouse with and without lymphadenitis respectively. Masses of bacteria, indicated by green arrowheads, stained dark brown by IHC and blue by H&E. Red arrowheads show tissue destruction in the sick mouse infected with the Δ*irp2* mutant. The lymph nodes (G, H and I) have an identical normal histology to uninfected lymph node [14]. Magnification, 40x (A, D, G, C, F and I) and 400x (B, E and H).

Six of the nine sick mice infected by fleabite with the wild-type strain were diagnosed with typical bubonic plague, characterized by severe lymphadenitis with destruction of the lymph node architecture and the presence of numerous bacteria admixed with cellular debris (Fig. 2A to 2C). The other three mice did not have obvious lymphadenitis, but the spleen and blood of all nine mice were heavily colonized (8.7±1.2 and 6.9±0.9 log_10_ CFU per ml of blood and spleen respectively). From these results one can infer that six mice had bubonic plague followed by sepsis and 3 mice developed primary septicemic plague (Table 1). In contrast, disease outcome in mice bitten by fleas infected with the *irp2* mutant was significantly different (*P* = 0.0015 by Fisher's exact test). Neither of the two mice that developed terminal disease after being bitten by fleas infected with the *irp2* mutant had typical bubonic plague (Figure 2). Histologic analyses of the lymph nodes proximal to the flea bite site of these mice did not reveal any bacteria (Fig. 2F and 2I), but the mouse that developed terminal plague at 6 days had evidence of lymphadenopathy (Figure 2D and 2E). Lymph node pathology was localized; however, many immature lymphocytes and macrophages containing ingested apoptotic lymphocytes were present throughout the entire lymph node. The etiology of this lymphadenitis is uncertain. It could have resulted from hematogenous spread subsequent to primary septicemic plague; alternatively, the lymph node may have been initially colonized and the bacteria disseminated to the blood before being eliminated from the node, in an atypical form of bubonic plague. Regardless of lymph node histopathology, both mice had a high bacterial load in the blood and the spleen (Table 1) (8.3 and 8.6 log_10_ CFU in the spleen and 3.8 and 7.6 log_10_ CFU per ml of blood). Hence, one mouse infected with the *irp2* mutant had primary septicemic plague, and the other had septicemic plague associated with a mild lymphadenitis. For mice challenged with either wild-type or Δ*irp2 Y. pestis*, disease outcome (bubonic plague, primary septicemic plague, or no disease) did not correlate with the cumulative number of challenges or infective flea bites (*P*>0.05 by Fisher's exact test).

## Discussion

The chromosomal *pgm* locus is prone to relatively frequent deletions and internal rearrangements that result in loss of the Hms or pigmentation segment, the HPI containing the Ybt operons, or both [16]. In previous studies, these phenotypically Pgm^–^ or Ybt^–^ spontaneous mutants were genetically undefined, or if defined were engineered in *Y. pestis* strains lacking virulence factors such as the pH6 antigen and YopJ, making it difficult to delineate the contribution to virulence of non-Ybt related genes within the *pgm* locus [5], [9], [10]. Recently, a reconstructed wild-type strain was mutated in the *irp2* gene and found to be essentially avirulent [11]. We found similar results using an *irp2* mutant produced from a fully virulent strain. Altogether, the data indicate that loss of a single Ybt-synthetic enzyme in a fully virulent *Y. pestis* strain can account for the avirulence observed for Pgm^–^ or Ybt^–^ strains in bubonic plague infection models, and support the hypothesis that the Ybt system provides the iron required by the bacteria during the early steps of the infection. Nonetheless, it cannot be excluded that the *pgm* locus encodes other virulence factors required for bubonic production since it was recently shown that a *pgm* negative mutant had a greater loss of virulence than the Ybt biosynthetic mutant in mouse model of pneumonic plague [11].

The present results with a *Y. pestis irp2* mutant mirror our previous results with a *Y. pestis* plasminogen activator (*pla*) mutant [2]. Both mutants are avirulent by the ID route and fully virulent by the IV route, but cause plague at low incidence following fleabite, despite the fact that their LD50 by the ID route (>10^5^ CFU) is several orders of magnitude higher than the number of CFU transmitted by blocked fleas (median <100 CFU) [2], [17], [18]. We previously proposed that direct injection of bacteria into a dermal blood vessel during the flea bite can lead to primary septicemic plague, with no prior bubonic stage [2]. The data herein provides independent support for this model.

The extent to which atypical forms of plague are attributable to atypical strains rather than the host immune response is unknown, but non-pigmented *Y. pestis* strains are frequently isolated from natural sources, and have been associated with mild cases of human plague [12]. The unstable nature of the *pgm* locus indicates that Ybt^–^ and Pgm^–^ clones are generated spontaneously in nature with some regularity. These clones would be at a disadvantage because of their decreased transmissibility, but could persist for some time during an epidemic associated with high flea density. The recently described early-phase transmission by fleas might also be more conducive to the circulation of these clones [19]. Because blocked fleas are unable to ingest blood, they probe repeatedly, a behavior that enhances deposition of bacteria into the extravascular space of the dermis. In contrast, unblocked fleas take a normal blood meal during early phase transmission, suggesting that direct IV transmission would be more common.
